# A New Paradigm for Atrial Fibrillation Ablation in Obesity Focusing on Substrate Remodeling and Patient-Centered Outcomes

**DOI:** 10.31083/RCM47591

**Published:** 2026-04-09

**Authors:** Ying Cao, Aobo Gong, Zexi Li, Fanghui Li, Xianjin Hu, Bangjiaxin Ren, Wenjie Li, Yifan Zhou, Rui Zeng

**Affiliations:** ^1^Department of Cardiology, West China Hospital, Sichuan University, 610041 Chengdu, Sichuan, China

**Keywords:** atrial fibrillation, obesity, catheter ablation, pulsed field ablation, epicardial adipose tissue, quality of life, substrate modification, photon-counting computed tomography

## Abstract

The obesity and atrial fibrillation (AF) epidemics are inextricably linked and continue to increase worldwide, posing significant global health burdens. Epidemiological evidence has revealed that obesity is a powerful, independent, and modifiable risk factor for AF. Obesity directly creates a proarrhythmic substrate through a triad of synergistic pathways: chronic hemodynamic overload that induces left atrial enlargement, local paracrine and inflammatory effects of dysfunctional epicardial adipose tissue (EAT), and systemic inflammation and oxidative stress, which collectively promote atrial fibrosis and electrical remodeling. Catheter ablation remains a cornerstone of rhythm control; however, the efficacy of this procedure is often compromised in patients with obesity, creating a “high-recurrence–high-benefit” paradox in which this patient group achieves the greatest improvements in quality of life despite higher rates of arrhythmia recurrence. Recent large-scale data have further refined this paradox, identifying a distinct “efficacy cliff” among patients categorized as severely obese (body mass index ≥35 kg/m^2^). Furthermore, emerging concepts are reshaping therapeutic strategies. Novel technologies, such as pulsed field ablation, with an enhanced safety profile, can mitigate the “insulation effect” of EAT; however, these concepts cannot fully overcome this effect without complementary strategic adjustments. Concurrently, recent evidence has suggested that ablation may act as a “biological substrate modification” by reducing the volume of local EAT. These findings support a paradigm shift in therapeutic strategy, moving beyond the singular endpoint of arrhythmia elimination toward a comprehensive approach that applies substrate modification guided by dynamic, spatiotemporally discrete mapping, with the primary endpoints of success shifting to dual endpoints encompassing a reduction in AF burden and an improvement in the quality of life of patients. Overall, this review aimed to discuss the pathophysiological nexus between obesity and AF, critically evaluate the challenges and technological advancements in catheter ablation for this population, and propose an integrated management pathway centered on substrate reversal and quality of life.

## 1. Introduction

Atrial fibrillation (AF) and obesity are two interconnected global public health challenges, with increasing prevalence contributing to a significant and growing disease burden [1, 2, 3]. AF is the most common sustained cardiac arrhythmia in clinical practice. It is driven by an aging population and the increasing prevalence of risk factors, such as obesity, hypertension, and diabetes [4], and its impact is expanding rapidly; in Europe, AF prevalence is projected to nearly double between 2016 and 2060 [2, 5, 6]. Concurrently, the global obesity epidemic continues unabated. The worldwide prevalence of obesity has nearly tripled since 1975, with the age-standardized rate rising from 4.6% in 1980 to 14.0% in 2019 [7].

The parallel rise of these two conditions is not coincidental. Robust epidemiological evidence confirms that obesity is not only a common comorbidity of AF but a powerful, independent, and modifiable risk factor deeply involved in the initiation, progression, and recurrence of arrhythmia [2, 8]. Obesity is the second leading attributable risk factor for AF, after hypertension [9]. Approximately 20% of individuals who are overweight or obese are likely to develop AF [10]. This risk demonstrates a clear dose-response relationship: for every 5-unit increase in body mass index (BMI), the risk of incident AF increases by 28% [11].

The strong epidemiological and pathophysiological links between obesity and AF make the development of effective treatment strategies for this population a clinical priority. Catheter ablation remains a cornerstone of rhythm control. The recent PRAGUE-25 randomized controlled trial, which directly compared catheter ablation to intensive lifestyle intervention combined with antiarrhythmic drugs in patients with obesity and AF, confirmed the superiority of ablation for maintaining sinus rhythm [12]. Furthermore, novel technologies such as high-power short-duration ablation [13] and pulsed field ablation (PFA) [14] can improve efficacy and safety in this group. Emerging evidence suggests that ablation energy may directly modulate atrial epicardial adipose tissue (EAT) [15].

However, the critical “obesity-AF ablation paradox” requires attention. Patients with obesity, characterized by complex atrial substrate remodeling and extensive EAT infiltration, are one of the groups with the highest risk of AF recurrence post-ablation. This risk is particularly pronounced in the morbidly obese subpopulation [16], where the absolute volume of EAT acts as a biological driver of AF and a physical barrier to effective energy delivery [17]. Despite their heavy baseline symptom burden and severely impaired health-related quality of life (QoL), this patient group often experiences the most profound improvements in symptoms and QoL after the procedure [18]. This “high-recurrence, high-benefit” contradiction underscores the limitations of a treatment framework that defines success primarily by the complete elimination of arrhythmia.

In this review, we aimed to systematically elucidate the pathophysiological mechanisms linking obesity and AF, critically examine the evidence, technical challenges, and advancements in catheter ablation for obese patients, and propose a redefinition of procedural success. We advocate a paradigm shift from traditional rhythm control towards a model that prioritizes atrial substrate modification and patient-centered outcomes, including symptom relief and quality of life. We define this “new paradigm” as an integrated strategy that combines the dynamic targeting of functional substrates and biological modulation of EAT with advanced energy sources, such as PFA, while prioritizing the reduction of symptom burden over the traditional binary judgment of recurrence. By integrating current evidence, we seek to construct a comprehensive, individualized clinical management framework to optimize outcomes for patients with obesity and AF.

## 2. The Pathophysiological Nexus: How Obesity Forges and Sustains the Atrial Fibrillation Substrate

**Fig. 1.**
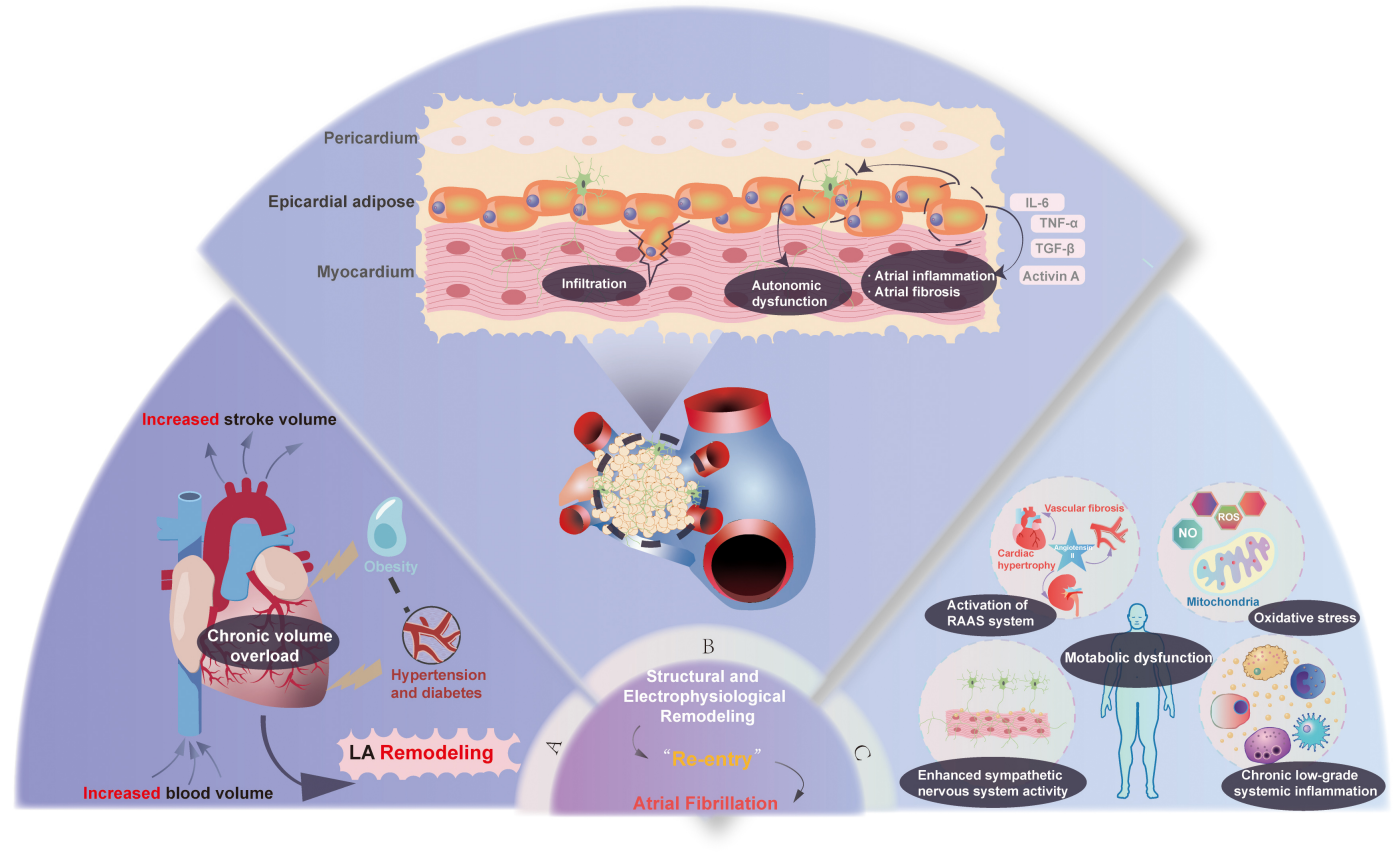
**The synergistic pathophysiology of obesity-driven atrial fibrillation, mediated by mechanical stress, local biochemical erosion, and systemic metabolic derangement**. This schematic illustrates the three interconnected pathways through which obesity promotes the development of an arrhythmogenic substrate for AF. These pathways are (A) mechanical stress and hemodynamic burden, (B) local biochemical erosion centered on epicardial adipose tissue (EAT), and (C) systemic biochemical and metabolic dysfunction. These mechanisms converge on a final common pathway of adverse atrial structural and electrophysiological remodeling, which initiates and sustains AF. LA, left atrial; AF, atrial fibrillation; RAAS, renin-angiotensin-aldosterone system; IL-6, interleukin-6; TNF-α, tumor necrosis factor-α; TGF-β, transforming growth factor-β. The figure was created by Adobe Illustrator 2025 (Adobe Inc.; San Jose, CA, USA).

Obesity systemically remodels the atrial structure and function through multiple interconnected pathways, creating and perpetuating a pro-arrhythmic substrate conducive to AF initiation and maintenance. This process is driven by three synergistic pathological pathways: mechanical stress and hemodynamic load [19, 20], local biochemical erosion [19, 20], and systemic biochemical derangements [19, 20, 21, 22, 23]. These forces collectively drive significant structural and electrical remodeling, forming the core foundation of obesity-related AF (Fig. 1).

### 2.1 Mechanical Stress and Hemodynamic Burden

In the obese state, the body’s total blood volume and cardiac output increase to meet elevated metabolic demands, thereby subjecting the heart to chronic volume overload [19, 24]. This altered hemodynamic stress increases left ventricular and left atrial (LA) pressures, promoting LA enlargement [25, 26], which is a well-established precursor to AF. Furthermore, obesity is frequently accompanied by metabolic abnormalities, including hypertension, insulin resistance, and diabetes, which further exacerbate the hemodynamic load and create cardiovascular strain [27].

### 2.2 Local Biochemical Erosion

EAT is a key player in the pathogenesis of obesity-related AF, acting as a critical local driver of arrhythmogenesis and not merely a passive fat depot. EAT is a visceral fat layer directly covering the atrial myocardium, sharing its microcirculation with no fascial barrier [28], allowing for a direct, intense, and localized influence on the atria. In obesity, EAT undergoes a dysfunctional hypertrophic transformation, shifting from a simple energy storage depot to a highly active endocrine and paracrine organ [29]. It secretes high concentrations of pro-inflammatory cytokines, such as interleukin-6 [30] and tumor necrosis factor-α [31], and pro-fibrotic adipokines like Activin A [32]. This local inflammatory and fibrotic milieu becomes a primary driver of atrial inflammation, fibrosis, and electrophysiological abnormalities. Moreover, adipocytes from the EAT can physically infiltrate the myocardial layer, separating cardiomyocytes [30] and disrupting normal electrical conduction pathways. EAT is also richly innervated with ganglionated plexi, and its expansion and inflammation can contribute to cardiac autonomic nervous system dysfunction [30], further increasing arrhythmic susceptibility.

### 2.3 Systemic Biochemical Derangement

Beyond the localized effects of EAT, obesity fosters a state of chronic [33, 34], low-grade systemic inflammation and oxidative stress that continuously promotes the deterioration of the atrial substrate. Circulating inflammatory mediators and reactive oxygen species act on the entire atrium, accelerating remodeling and reducing the electrical stability of cardiomyocytes, lowering the threshold for ectopic triggers to initiate AF [35].

Obesity also leads to enhanced sympathetic nervous system activity and activation of the renin-angiotensin-aldosterone system [36]. These neurohormonal changes contribute to increased vascular resistance, atrial stretch [35], and direct pro-fibrotic effects [37, 38] on the atrial myocardium. Concurrently, obesity-related metabolic disturbances, such as insulin resistance and elevated free fatty acid levels, induce further oxidative stress, cardiomyocyte lipotoxicity, and mitochondrial dysfunction [39], thereby compromising electrophysiological stability.

### 2.4 The Final Common Pathway: Vicious Cycles of Structural and Electrophysiological Remodeling

The long-term interplay of mechanical load as well as local and systemic biochemical challenges culminate in profound and deleterious structural and electrical remodeling of the atria. Structural remodeling is the central feature of obesity-related AF, manifesting primarily as progressive LA enlargement, interstitial fibrosis, and fatty infiltration of the atrial myocardium [19]. These structural changes disrupt the normal architecture and cell-to-cell connections of the atria by causing the lateralization of gap junction proteins, such as Connexin-40, which are crucial for coordinated electrical signaling [40].

This structural disarray inevitably translates into electrophysiological dysfunction. Atrial fibrosis and fatty infiltration create obstacles and tortuous pathways for electrical impulses, slowing conduction velocity and increasing heterogeneity of conduction across the atria [41]. This slow and non-uniform conduction provides the ideal substrate for the formation of re-entry circuits, which are the core mechanism responsible for sustaining AF. Electrophysiologically, this is manifested as increased fractionated electrograms and low-voltage areas (LVAs) on electroanatomic mapping [42].

## 3. Catheter Ablation in Obesity: Unique Challenges and Strategic Considerations

Catheter ablation has become a cornerstone of rhythm control therapy for AF; however, its application in the obese population is fraught with a unique set of challenges that affect procedural safety, execution, and long-term efficacy.

### 3.1 Procedural Complexities and Suboptimal Efficacy

From a procedural standpoint, patients with obesity present numerous complexities. In terms of anesthesia and perioperative management, these patients frequently have comorbidities, such as obstructive sleep apnea (OSA), which complicates airway management. Altered pharmacokinetics can affect drug dosing, and compromised respiratory function significantly increases anesthetic risk [43]. In addition, some evidence suggests that these patients may experience greater pain perception during ablation procedures performed under conscious sedation [44], necessitating individualized management by an experienced anesthesia team.

The technical aspects of the procedure are also more challenging. Increased thickness of subcutaneous fat can obscure anatomical landmarks, making vascular access through the femoral vein harder. Post-procedural hemostasis is also harder to achieve, leading to a higher incidence of vascular complications, such as hematomas and pseudoaneurysms [45]. One study reported a significantly higher rate of vascular complications in patients with morbid obesity compared to their non-obese counterparts [46].

Furthermore, the body habitus in patients with obesity can degrade the quality of essential imaging modalities [47], including intracardiac echocardiography, transthoracic echocardiography, and computed tomography (CT), which are vital for pre-procedural planning and intra-procedural guidance. This reduction in image quality can lead to longer procedure times and significantly increase patient and operator exposure to X-ray fluoroscopy and its associated radiation dose [48].

Beyond these procedural challenges, growing evidence consistently demonstrates that a higher BMI is an independent predictor of AF recurrence following catheter ablation [49]. This risk is dose-dependent, with recurrence rates increasing with higher BMI [49, 50]. In patients with morbid obesity, the 3-year recurrence rate can be as high as 48% [50]. This negative impact of BMI on ablation outcomes persists even after adjusting for confounding comorbidities, such as OSA [51]. This persistent recurrence gap indicates that standard pulmonary vein isolation (PVI) alone is insufficient to address the diffuse and extensive substrate remodeling driven by the obesity-inflammation axis.

### 3.2 The Evolution of Substrate Modification: From Empirical Failures to Individualized Success

Given that obesity drives extensive atrial structural and electrical remodeling that often extends beyond the pulmonary veins (PVs) [2, 19], there is a strong theoretical rationale for employing more extensive substrate modification strategies in addition to standard PVI. This advanced pathological substrate can manifest as LVAs, and mapping and ablation of these areas represents a key substrate-modification strategy beyond PVI [52]. Notably, some studies have shown that patients with obesity have more extensive LVAs [53], with a distribution that correlates with regions of EAT deposition [54], providing a basis for adding LVA ablation to PVI to reduce recurrence in this subgroup. Moreover, since patients with obesity are more likely to develop persistent AF [55], a condition known to respond poorly to PVI alone, a more aggressive substrate-based approach seems logical.

However, early attempts to translate this theoretical rationale into clinical benefit were met with challenges that generated significant controversy. Initial substrate modification strategies primarily relied on static identification of LVAs, the subjective targeting of complex fractionated atrial electrograms (CFAEs), or empirical anatomical linear ablation. Landmark randomized trials, including STAR AF II and STABLE-SR-II, yielded neutral results [56, 57], robustly demonstrating that the routine addition of LVA ablation provided no incremental benefit. The crucial lesson from these studies was not the failure of the “substrate modification” concept, but rather the failure of the specific targets. Mapping of LVAs and CFAEs is highly subjective and poorly reproducible, potentially identifying mere “bystanders” of atrial pathology rather than genuine arrhythmogenic “drivers”. This highlighted the inherent limitations of traditional, static voltage mapping in identifying the functionally relevant arrhythmic substrate [58].

The period of controversy ultimately underscored a critical unmet need: an objective, reproducible method for identifying functionally pertinent substrate targets. Notably, the TAILORED-AF trial by Deisenhofer *et al*. [58], the first large, multinational, randomized and superiority-controlled trial of its kind, successfully addressed this by demonstrating that a “PVI-plus” strategy, guided by a dynamic, precise, and reproducible artificial intelligence (AI) algorithm, was significantly superior to PVI alone in patients with persistent AF. This study marks a major paradigm shift by directly addressing the limitations of previous trials. Unlike the static delineation of LVAs, the AI in TAILORED-AF analyzed dynamic conduction patterns in real time to identify spatiotemporal dispersion zones suggestive of localized re-entrant conduction, a mechanism closer to the core sustenance of AF, which was pivotal to the trial’s success.

Notably, 43% of the 370-patient cohort in the TAILORED-AF trial had obesity (BMI >30 kg/m^2^). Consequently, the 88% success rate of the AI-guided strategy was achieved in a population where nearly 50% the patients had obesity [58]. This provides powerful corroborating evidence for our central thesis that patients with obesity, who represent a complex substrate phenotype, require individualized substrate modification beyond PVI.

Therefore, optimal clinical practice should be guided by an individualized approach. The final decision on ablation strategy should be based on a dynamic and meticulous intraprocedural assessment of the functional atrial substrate, leveraging advanced technologies, such as AI, rather than being predetermined solely by the patient’s BMI or by using static voltage maps.

### 3.3 A New Dawn in Technology: PFA and the Challenge of the “Dielectric Barrier”

PFA is a revolutionary non-thermal ablation technology that offers unprecedented hope for overcoming the unique pathophysiological challenges faced in patients with obesity. PFA delivers high-voltage, microsecond-duration electrical pulses to induce irreversible electroporation in cardiomyocyte membranes, leading to cell death [59]. This mechanism is highly tissue-selective for myocardium [59], which has a lower threshold for electroporation than surrounding tissues, such as the esophagus or phrenic nerve, thereby dramatically reducing the risk of collateral damage. As a “single-shot” technology, PFA can significantly shorten procedure times [60]. PFA has been shown to reduce contrast dye usage and radiation exposure in patients who are overweight and those with obesity [61]. However, the application of PFA in the obese population requires a critical re-evaluation of its physical interaction with the unique atrial substrate.

The success of conventional thermal ablation (radiofrequency or cryoballoon) depends heavily on the efficient conduction of energy through tissues to create a sufficiently deep and transmural lesion. In patients with obesity, the thickened layer of EAT presents a formidable physical barrier. Adipose tissue has very low electrical and thermal conductivity; therefore, it acts as an “insulation layer”, severely impeding the effective transfer of thermal energy [17]. This leads to shallow, non-transmural lesions, which is a key pathophysiological reason for the high recurrence rates observed in these patients.

The advantage of PFA lies in its non-thermal mechanism. Electroporation occurs when the electric field strength applied across a cell membrane reaches a critical threshold, irrespective of tissue temperature. Preclinical studies have confirmed that PFA’s electric field can effectively penetrate fat layers and fibrous collagen, creating uniform, transmural lesions in deep myocardial tissue often unreachable with traditional thermal energy [59]. One comparative study clearly demonstrated that radiofrequency ablation effects were largely confined to the endocardial surface in scarred tissue with intervening fat and collagen, whereas PFA could penetrate these barriers to achieve complete, transmural ablation from endocardium to epicardium [62]. Mechanistically, PFA is uniquely positioned to bypass the “insulation effect” of EAT, offering a strong theoretical basis for achieving more durable and reliable PVI and superior clinical outcomes in patients with obesity. However, biophysical modeling and clinical data suggest a more complex reality dominated by “dielectric shielding”. PFA circumvents the “heat sink” phenomenon that compromises thermal ablation, but it confronts the high electrical impedance of adipose tissues. The electrical conductivity of adipose tissue (σ ≈ 0.03 S/m) is significantly lower than that of the myocardium (σ ≈ 0.60 S/m) [63]. Thus, in patients with obesity, thick layers of EAT can attenuate electric field strength by shunting current laterally, potentially preventing the formation of a transmural voltage gradient sufficient to induce irreversible electroporation in the sub-epicardial myocardium.

Emerging clinical evidence is still in its early stages and presents a mixed picture. A propensity-matched study suggested that PFA provided superior rhythm control compared with cryoballoon ablation in patients with obesity and AF [60]. In contrast, a retrospective study revealed that the 1-year AF-free survival rates were comparable between PFA and cryoballoon ablation in patients who are overweight or have obesity [61]. Another study observed that even after PFA, each unit increase in BMI was associated with a 4.2% increase in the risk of AF recurrence [64]. This large-scale registry (the EU-PORIA registry, n = 1055) data corroborates these biophysical constraints, revealing a distinct “efficacy cliff” in patients with severe obesity; it identified a critical threshold at a BMI of 35 kg/m^2^ [64]. Specifically, while PFA maintained high efficacy in patients who are overweight or have Class I obesity, those with severe obesity (BMI ≥35 kg/m^2^) exhibited a statistically significant increase in arrhythmia recurrence (37.2% at 1 year) [64]. Furthermore, recent re-mapping studies (Najafi *et al*. [65]) have challenged the assumption of durable isolation in this subgroup, showing significantly lower rates of durable PVI in patients with severe obesity than in normal-weight controls.

This inconsistency in data likely stems from the following factors. Patient populations are heterogeneous across studies. The most promising results have been observed in patients who are overweight or have Class I obesity. In those with severe obesity (BMI ≥35 kg/m^2^), the extremely thick EAT and more advanced atrial substrate remodeling may still pose a significant challenge to current PFA technologies and energy delivery protocols. Crucially, electrical reconnection was predominantly localized to anatomical segments associated with high EAT burden, such as the left atrial roof and posterior wall [66, 67]. This validates the hypothesis that thick adipose tissues act as a dielectric shield, causing electric field attenuation. Consequently, cardiomyocytes in these high-impedance regions may undergo reversible electroporation (“stunning”) rather than irreversible cell death, creating a substrate for late electrical reconduction and AF recurrence.

In summary, the inconsistencies in current data can be attributed to the significant heterogeneity in study populations and the variable impact of EAT thickness, which acts as a distinct “dielectric” barrier. This underscores the urgent need for large-scale, prospective randomized controlled trials specifically designed to compare PFA with traditional thermal ablation across different strata of obesity (BMI 30–35, 35–40, and >40 kg/m^2^). Such trials should incorporate obesity-specific energy titration studies and systematic re-mapping procedures to assess lesion durability, which will be essential for defining the precise role and optimal application strategy for PFA in this large and growing patient population.

### 3.4 Imaging Frontiers: Advanced Substrate Evaluation and Pre-Procedural Planning With Cardiac Magnetic Resonance and Photon-Counting Computed Tomography

The transition of AF ablation from a strategy focused solely on PVI to a “PVI-plus” substrate-modification approach requires precise quantification of the arrhythmogenic substrate (EAT and fibrosis) for its scientifically grounded execution. In this context, cardiac magnetic resonance (CMR) and photon-counting computed tomography (PCCT) represent cutting-edge clinical tools for this advanced assessment.

#### 3.4.1 CMR: The Gold Standard for Tissue Characterization

The principal strength of CMR lies in its unparalleled capability for non-invasive tissue characterization. Its late gadolinium enhancement technique serves as the clinical reference standard for identifying atrial fibrosis [68]. More critically, CMR offers unique insights into the quality of EAT. Using T1 mapping, CMR can non-invasively probe the inflammatory and metabolic activity of EAT [69]. Given that the pro-inflammatory paracrine effects of EAT constitute a core mechanism in obesity-related AF, CMR provides an indispensable biological perspective on the substrate.

#### 3.4.2 PCCT: A Resolution Revolution and Precise EAT Quantification

While CMR offers effective qualitative assessment, its spatial resolution limitations make measurements of thin EAT layers susceptible to partial volume effects (PVE), often leading to volume over- or underestimation [70]. PCCT, with its energy-resolving detectors that eliminate electronic noise, achieves ultra-high spatial resolution at the sub-millimeter level [71]. This technological leap fundamentally mitigates PVE, enabling the precise delineation of even very thin EAT layers from adjacent myocardium [72]. Notably, several studies confirm that PCCT surpasses conventional CT and CMR in the accuracy and reproducibility of EAT volume quantification, establishing it as the most precise morphological tool for monitoring subtle structural remodeling following weight loss or ablation [71, 72, 73].

#### 3.4.3 Towards an Integrated “One-Stop” Efficient Workflow

From a clinical translation standpoint, PCCT demonstrates significant potential to replace the traditional and fragmented pre-procedural workup (typically involving transesophageal echocardiography (TEE) plus CT/CMR) with an integrated “one-stop” imaging solution. First, the high resolution of PCCT achieves exceptional sensitivity for detecting left atrial appendage thrombus [74], demonstrating the potential to become a non-invasive alternative to TEE for thrombus surveillance. Second, a single PCCT acquisition can simultaneously provide detailed PV anatomy, accurate EAT volumetry, and myocardial extracellular volume fraction data that correlates highly with CMR-derived fibrosis assessment [75], thereby yielding a comprehensive substrate map.

We acknowledge that the widespread adoption of CMR is limited by scanner availability and expertise. Therefore, although CMR remains indispensable for analyzing tissue biological characteristics, PCCT, with its superior spatial resolution, minimized partial volume effects, and ability to consolidate anatomical evaluation, precise EAT quantification, and thrombus exclusion into a single, rapid scan, offers a more time-efficient, cost-effective, and patient-friendly pre-procedural planning solution for patients with obesity and AF. To ground our proposal in clinical reality, we emphasize a stratified approach where advanced substrate assessment is prioritized for patients with BMI ≥35 kg/m^2^, to whom the cost of advanced imaging may be justified by the reduction in redo procedures and improved long-term rhythm control. To the contrary, for lower-risk patients (BMI <30), standard care (Echo/CT) remains sufficient.

## 4. A Biological Treatment Opportunity: The Direct Impact of Ablation on EAT

Beyond efforts to refine surgical strategies to counteract the obese substrate, recent research has unveiled a fascinating possibility: that catheter ablation may exert a direct therapeutic effect on the EAT, the “fuel” for AF, thereby playing a previously unrecognized role in biological substrate modification.

### 4.1 Evidence for Post-Ablation EAT Reduction

A landmark CMR study provided compelling evidence for this phenomenon. In a systematic imaging assessment of 15 patients before and after catheter ablation, researchers observed a striking reduction in left atrial EAT volume. The median left atrial EAT volume decreased from 35.2 mL pre-ablation to 16.2 mL post-ablation, representing a relative reduction of 46% (*p* < 0.001) [15]. This significant change was observed in patients treated with radiofrequency and cryoballoon ablation and occurred in the context of a stable BMI, strongly suggesting that the EAT reduction was a direct or indirect consequence of the ablation procedure, not a secondary effect of systemic weight loss. However, the limited sample size (n = 15) necessitates that these findings be interpreted as generating a conceptual framework rather than a definitive conclusion. The precise pathway linking ablation to EAT reduction remains a critical open question awaiting further validation.

### 4.2 Potential Mechanisms of EAT Regression

The precise mechanisms underlying this effect are yet to be fully elucidated; however, several plausible explanations exist. First, the energy delivered during ablation may directly injure or induce apoptosis in adjacent adipocytes [15]. Second, radiofrequency ablation causes microvascular endothelial damage that extends beyond the visible lesion border [76]; this disruption of the microcirculation could compromise the blood supply to the EAT, promoting atrophy [15]. Third, post-ablation CMR T2-weighted imaging reveals widespread signal hyperintensity indicative of a local inflammatory response [77], and this altered inflammatory microenvironment may modulate fat metabolism and remodeling processes. Finally, the restoration of sinus rhythm or a significant reduction in AF burden can lead to EAT reduction [78], possibly through secondary effects such as improved atrial hemodynamics and reduced wall tension, which may promote reverse remodeling of the EAT.

### 4.3 Transformative Clinical Implications: From “Building a Firewall” to “Removing the Fuel”

This discovery suggests that catheter ablation may possess a dual therapeutic value, combining “electrical isolation” and “biological modification”. The procedure creates scar tissue to establish an electrical conduction block and favorably remodels the biological substrate by reducing EAT, thereby directly attenuating the local pro-arrhythmic microenvironment.

This finding could fundamentally alter our understanding of the mechanism of action of catheter ablation. If EAT acts as the metabolic ‘fuel’ for the AF ‘fire’ [79], then ablation transcends its traditional role of merely building a ‘firewall’ (electrical isolation) to actively ‘removing the fuel’. This allows for the reconceptualization of catheter ablation as a targeted “biological substrate modification” that directly attenuates the local, pro-arrhythmic microenvironment. This may be a critical, previously unrecognized mechanism contributing to its success. Notably, several pieces of evidence support this approach. Chamoun *et al*. [15] observed a striking 46% reduction in left atrial EAT volume post-ablation that occurred independently of systemic weight loss, implying a direct therapeutic injury to local adipocytes. Moreover, as mentioned above, the drivers of this ‘de-fueling’ process appear to be multi-dimensional. In larger cohorts, Watanabe *et al*. [78] and Shimojo *et al*. [80] demonstrated that sustained EAT regression was predominantly confined to patients maintaining sinus rhythm, whereas arrhythmia recurrence was accompanied by EAT re-expansion and ganglionated plexi reactivation. The latter further elucidated the potential inflammatory mechanisms associated with EAT, reinforcing the biological plausibility of targeting adipose tissue. Collectively, these findings suggest that ‘removing the fuel’ is a synergistic outcome of acute ablative injury and chronic reverse remodeling following hemodynamic unloading. This insight opens up a new dimension for developing next-generation ablation technologies. To fully realize its translational potential, distinguishing the relative contributions of direct ablative injury versus secondary hemodynamic unloading remains a critical frontier for future mechanistic verification.

Based on this new understanding of EAT regression, one can speculate that future PFA technologies could be optimized by adjusting pulse voltage, width, or frequency to achieve precise cardiomyocyte electroporation and maximize the induction of adipocyte apoptosis, thereby maximizing the beneficial EAT reduction effect. This implies that the long-term success of ablation may depend as much on the biological response (the degree of EAT regression and inflammation reduction) as on the acute electrical result. Future clinical trials should focus on the durability of isolation and systematically evaluate the impact of different energy parameters on EAT volume and function, perhaps using serial PCCT to monitor post-ablation EAT changes as a potential biomarker for predicting long-term success.

## 5. The Patient-Centered Imperative: QoL as an Essential Outcome

In AF management, patients are often concerned with improving subjective symptoms and functional status. At this level, catheter ablation offers another crucial opportunity for patients with obesity and AF.

### 5.1 The Dual Impact of AF and Obesity on Health-Related QoL

Symptomatic AF severely impairs a patient’s QoL, with QoL scores comparable to those of patients with moderate heart failure [81]. Obesity exacerbates this burden, independently lowering QoL [81], particularly in the domain of physical health. Studies have shown that compared to patients with AF having a normal weight, those with a BMI ≥30 kg/m^2^ report lower scores on nearly all subscales of the SF-36 health survey [82]. This combination results in a patient population with a particularly heavy symptom burden and an extremely poor baseline QoL [81].

### 5.2 Disproportionate QoL Gains in Patients With Obesity Post-Ablation

Catheter ablation can produce substantial and positive changes in QoL for patients with AF [81, 82, 83]. Validated instruments, such as the generic Medical Outcomes Study 36-item short-form health survey (SF-36) [18, 81, 82, 83] and the disease-specific Atrial Fibrillation Effect on Quality-of-Life (AFEQT) questionnaire [84], can quantify this improvement. For example, a multicenter cohort study demonstrated that AFEQT scores improved significantly across all weight groups post-ablation. Notably, while patients with obesity started with lower pre-procedural scores, their scores improved to 81 post-ablation, and although this was still slightly lower than the scores for patients with normal weight or those who are overweight, the magnitude of the improvement was profound [84]. A meta-analysis further highlighted that despite having lower baseline QoL scores, the QoL gap between patients with high BMI and normal BMI narrowed significantly after ablation [18]. This indicates that patients with obesity derive a disproportionately large QoL benefit from the intervention.

### 5.3 The Decoupling of QoL Improvement From Absolute Rhythm Control

A critically important finding is that the improvement in QoL is not entirely dependent on the absolute maintenance of the sinus rhythm. Even when arrhythmia recurrence is documented post-ablation, patients can still experience a significant improvement in QoL, although the degree of improvement is greatest in those who remain free of AF [85]. This suggests that reducing the overall AF burden is a valuable and clinically meaningful therapeutic goal. This supports the establishment of symptom relief and functional recovery as vital endpoints in the management of AF in patients with obesity [50].

This decoupling of QoL from perfect rhythm control fundamentally changes the definition of procedural success. It validates a strategy of “AF burden reduction” rather than “AF elimination” and provides a strong ethical and clinical justification for intervention even when the chance of a complete “cure” is lower. For patients with obesity who are highly symptomatic, the profound and reliable improvement in QoL often heavily outweighs the higher risk of recurrence [18, 38, 50, 83, 86]. This observation demands a reframing of the risk-benefit analysis and the informed consent process. The therapeutic goal should be explicitly defined as “alleviating symptom burden and improving quality of life”. This approach allows for a more rational assessment of the benefits of the procedure and provides a more patient-centered therapeutic option for this population. In addition, this has significant implications for healthcare policy, suggesting that future clinical trials and reimbursement decisions for ablation in this population should incorporate validated QoL instruments as primary or key secondary endpoints.

## 6. The Cornerstone of Comprehensive Therapy: Weight Management as a Substrate Reversal Strategy

Given that obesity is the central engine driving the remodeling of the atrial substrate in AF [87], any therapeutic strategy aimed at long-term success must focus on weight management. Far from being a simple lifestyle recommendation, weight management is a powerful, non-invasive therapy capable of directly reversing the pro-arrhythmic atrial substrate [54, 78].

### 6.1 Foundational Role of Lifestyle Intervention

Structured lifestyle intervention forms the foundation of weight management. Physician-led programs that incorporate structured dietary and exercise guidance have been proven to significantly reduce AF burden and slow disease progression [88, 89]. The landmark LEGACY study provided definitive evidence for this, demonstrating that patients who achieved and maintained more than 10% weight loss had a six-fold greater probability of arrhythmia-free survival compared to those who lost less than 3% of their body weight [89]. This highlights the profound and lasting impact of sustained weight control on atrial electrophysiological stability.

### 6.2 Adjunctive Pharmacological and Surgical Interventions

For many patients, lifestyle changes alone may be insufficient, necessitating the use of more intensive adjunctive therapies. Glucagon-Like Peptide-1 receptor agonists (GLP-1 RAs) have emerged as a new class of highly effective weight-loss medications [90]. Their benefit in AF may extend beyond simple weight reduction, as they have been shown to preferentially reduce EAT and exert direct anti-inflammatory effects [91, 92], thereby directly improving the atrial substrate. However, data on their impact on post-ablation AF recurrence are currently inconclusive. Satti *et al*. [93] reported no benefit from preoperative use (hazard ratio [HR]: 1.04; *p* = 0.51); however, Patel *et al*. [94] demonstrated a significant reduction in recurrence risk among patients with obesity (HR: 0.72; *p* < 0.001). Consistently, Venier *et al*. [95] substantiated this protective association, reporting a comparable decline in arrhythmia recurrence (HR: 0.82; *p* < 0.0001). These divergent findings likely reflect heterogeneity in drug generation, timing of initiation, and study design, underscoring the imperative for prospective randomized trials to clarify the role of GLP-1 RAs in this setting.

For patients with morbid obesity, bariatric surgery offers the most profound and durable weight loss [96]. The impact of this intervention on the atrial substrate is remarkable. Studies have shown that the post-ablation recurrence rate is dramatically reduced in patients with morbid obesity who undergo bariatric surgery prior to AF ablation, falling from approximately 61% to around 20% [97]. This suggests that bariatric surgery can effectively “normalize” the atrial substrate, bringing the patient’s procedural risk down to a level comparable to that of the non-obese population. This dramatic effect provides a message of profound hope, demonstrating that the obesity-related atrial substrate is, to a significant degree, reversible.

## 7. A Proposed Clinical Pathway for the Management of Atrial Fibrillation in Patients With Obesity

**Fig. 2.**
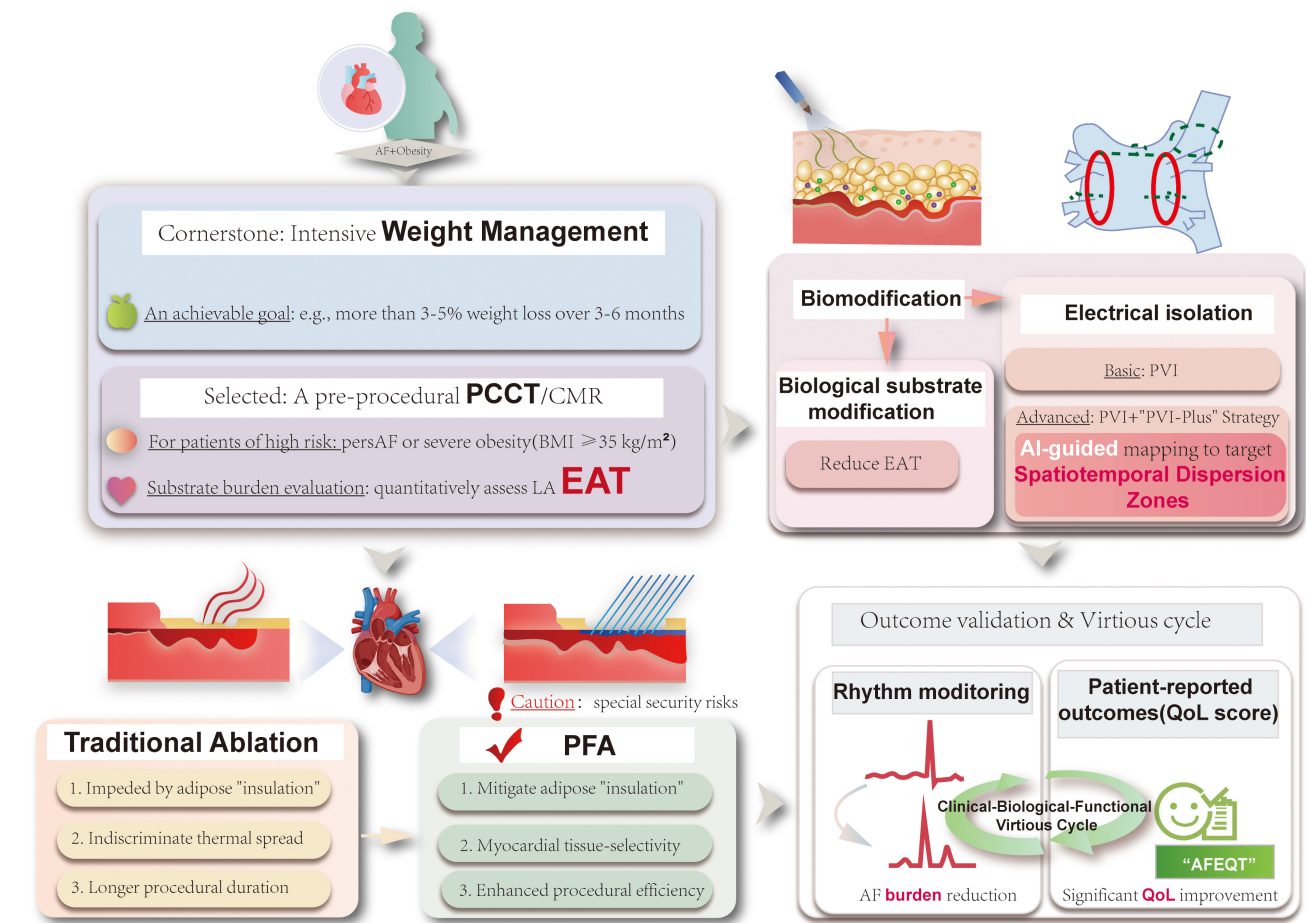
**An integrated, substrate-oriented management pathway for patients with obesity and AF**. This figure outlines a novel clinical paradigm that combines the dynamic targeting of functional substrates and biological modulation of EAT with advanced energy sources, such as PFA, while prioritizing the reduction of symptom burden over the traditional binary judgment of recurrence. PersAF, persistent atrial fibrillation; CMR, cardiac magnetic resonance; PCCT, photon-counting computed tomography; EAT, epicardial adipose tissue; PFA, pulsed field ablation; AI, artificial intelligence; “AFEQT”, “Atrial Fibrillation Effect on Quality-of-Life” (questionnaire); QoL, quality of life; PVI, pulmonary vein isolation. The figure was created by Adobe Illustrator 2025 (Adobe Inc.; San Jose, CA, USA).

Based on the available evidence, AF management in patients with obesity requires a multi-dimensional, integrated strategy. The core elements of this approach include aggressive pre-procedural weight management, advanced substrate assessment, individualized intra-procedural ablation strategies, and the adoption of QoL as a primary outcome measure. The following phased pathway is proposed to operationalize this new paradigm (Fig. 2).

In the pre-procedural phase, a structured weight management program should be initiated immediately upon diagnosis for any patient with obesity being considered for ablation, with a clear and achievable goal, such as achieving ≥3–5% weight loss over 3–6 months [87]. For selected patients, particularly those with persistent AF or severe obesity, pre-procedural PCCT or CMR can be considered to quantitatively assess LA EAT. This advanced substrate assessment helps identify individuals with a high arrhythmogenic burden, assisting in risk stratification and the planning of a personalized procedural strategy that may extend beyond simple PVI. For these high-risk patients, planning should involve the use of advanced electrophysiological mapping techniques such as AI-guided mapping to precisely identify and target potential functional driver mechanisms or spatiotemporal dispersion zones.

During the intra-procedural phase, the pre-planned, individualized strategy should be executed with precision. The choice of ablation technology is paramount, and PFA presents distinct advantages for this patient population. The primary technical goals are to achieve durable PVI and effective modification of any targeted substrate, while ensuring maximal patient safety. It must be acknowledged that PFA is not a universal solution. While it offers a superior safety profile by significantly reducing the risk of certain major complications associated with conventional thermal ablation, important safety considerations remain, including the potential for cardiac tamponade, coronary spasm, hemolysis-related acute kidney injury, and vasovagal reactions, all of which require continued clinical vigilance [98, 99, 100]. Consequently, caution is advised when employing PFA in specific scenarios, such as for linear lesions adjacent to coronary arteries [101]. In these high-risk anatomical situations, or for patients with known significant coronary artery disease, alternative energy sources like radiofrequency ablation may be more appropriate.

Following the procedure, comprehensive post-procedural management is critical for long-term success. This includes continued emphasis on and support for long-term weight management and the control of all relevant cardiovascular risk factors. Crucially, this new paradigm redefines therapeutic success by transcending simple arrhythmia recurrence. Evaluation should be based on dual metrics, incorporating standard rhythm monitoring to quantify AF burden reduction, and the systematic collection of QoL data using validated questionnaires. To operationalize patient-centered care, we recommend integrating the disease-specific AFEQT questionnaire, which mitigates the confounding ‘floor effect’ of obesity-related comorbidities inherent in generic scales [102]. Adherence to the ICHOM Standard Set is advised, mandating data collection at baseline, 6 months, and annually to capture long-term symptom durability beyond the blanking period [103]. Implementation should leverage automated Electronic Health Record digital triggers to minimize workflow disruption [104], utilizing the Minimal Clinically Important Difference of ±5 points as a threshold for clinical review [105]. This dual-endpoint approach ensures a comprehensive assessment of treatment success and facilitates the holistic improvement of outcomes in patients with obesity.

## 8. Limitations 

This article is limited by the heterogeneity of the available evidence. In particular, the role of PFA in patients with obesity remains to be defined by adequately powered prospective studies with comprehensive stratification across degrees of obesity. EAT reduction after ablation appears to have mechanistic and clinical relevance, although the underlying pathways and the extent of its contribution remain to be clarified. Additional prospective studies are needed to validate the emerging components of the proposed clinical framework.

## 9. Conclusions

This review systematically argues that for patients with obesity and AF, the standard for success in catheter ablation requires a fundamental paradigm shift. It is imperative to move beyond the traditional model focused solely on achieving freedom from arrhythmia and embrace a comprehensive new paradigm that integrates substrate modification, symptom control, and the enhancement of quality of life. Technologically, this demands a pivot to precision. AI-guided dispersion ablation provides a validated method for targeting functional substrates; however, the biophysical challenge of “dielectric shielding” in severe obesity necessitates adaptive, high-energy protocols for PFA to ensure lesion durability. Concurrently, the therapeutic focus must expand to include “biological substrate modification”, leveraging the capacity of ablation energy to induce the regression of pathogenic epicardial adipose tissue. Ultimately, clinical success must be redefined through dual metrics, which validate the profound symptomatic relief unique to this population, transforming high-risk care into a pathway for durable health improvement.

## Abbreviations

AF, atrial fibrillation; BMI, body mass index; CMR, cardiac magnetic resonance; CT, computed tomography; EAT, epicardial adipose tissue; LVAs, low-voltage areas; OSA, obstructive sleep apnea; PCCT, photon-counting computed tomography; PFA, pulsed field ablation; QoL, quality of life; LA, left atrial.
